# Effects of Endophytic Entomopathogenic Ascomycetes on the Life-History Traits of *Aphis gossypii* Glover and Its Interactions with Melon Plants

**DOI:** 10.3390/insects10060165

**Published:** 2019-06-10

**Authors:** Natalia González-Mas, Araceli Sánchez-Ortiz, Pablo Valverde-García, Enrique Quesada-Moraga

**Affiliations:** 1 Departamento de Agronomía, Universidad de Córdoba, ETSIAM, Campus Rabanales, Edificio C4 Celestino Mutis, E-14071 Córdoba, Spain; z22goman@uco.es (N.G.-M.); cr2vagap@uco.es (P.V.-G.); 2 IFAPA Ctr Venta del Llano, Ctra Bailen Motril, Km 18-5, 23620 Jaen, Spain; araceli.sanchez.ortiz@juntadeandalucia.es

**Keywords:** Biological control, *Beauveria bassiana*, *Metarhizium brunneum*, endophyte, pre-mortality effects, tritrophic interactions

## Abstract

Entomopathogenic fungi are sprayed commercially for aphid control in greenhouses. Recently, their ability to grow endophytically within plants was discovered, offering the opportunity for systemic biological control. Endophytic colonization of host plants could also influence life-table parameters and behavior of herbivores. We investigated lethal and pre-mortality effects of *Beauveria bassiana* and *Metarhizium brunneum* on *Aphis gossypii*; aphids either received inoculum while feeding on recently sprayed leaves (surface inoculum and endophytically-colonized) or while feeding on unsprayed but endophytically-colonized leaves. We used choice assays to identify any preferences for endophytically-colonized or control plants. Volatile emissions from endophytically-colonized plants and control plants were also compared. Aphid mortality rates ranged between 48.2 and 56.9% on sprayed leaves, and between 37.7 and 50.0 on endophytically-colonized leaves. There was a significant effect of endophytic colonization on the rate of nymph production, but this did not result in an overall increase in the aphid population. Endophytic colonization did not influence host-plant selection even though there were qualitative and quantitative differences in the blend of volatiles released by endophytically-colonized and control plants. Although endophytic colonization did not change herbivore behavior, plants still benefit via indirect defense, resistance to plant pathogens or abiotic stress tolerance.

## 1. Introduction

Controlling aphids is a challenge for growers because, not only do aphids cause damage to crops by direct feeding, but also by transmission of economically costly plant viruses [1]. Aphids also have extremely high fecundities and short developmental times, allowing populations to grow very rapidly [2]. For these reasons, control commonly relies on intensive use of systemic chemical insecticides which, coupled with their reproductive rate, has led to the emergence of widespread resistance to currently-available modes of action [3]. Entomopathogenic fungi are the most important microbial control agents naturally regulating aphid populations and, therefore, have great potential as biological control agents of these pests [4].

Most fungal species naturally infecting aphids are from the Entomophthoromycota and Ascomycota (order Hypocreales). Species in the Ascomycota have been commercialized for aphid control because they can be readily mass-produced in vitro and easily formulated for spray application [5]. Indeed, several mycoinsecticides for aphid control are available on the market and sold as environmentally-safe products that fit perfectly into current legislation requirements [6]. They can be applied by spraying, drenching or seed dressing [7,8], or can be established as transient endophytes in targeted crops where they protect the plants from pests [9,10,11].

Whilst the efficacy of transient endophytic colonization has been proven against some sucking insects, such as whiteflies [10], it should be noted that the immature developmental stages of these species are attached to the plant and do not move within and between plants. In contrast, all stages of aphids (except eggs) are mobile and probe the phloem to feed [12,13]. Hence, they could avoid contact with fungal propagules. For this reason, it is important to determine whether endophytic colonization of plants by entomopathogenic fungi can cause aphid mortality. It also remains unclear whether the conidia that remain coating the leaves and stems combined with endophytic colonization can affect aphid survival.

Furthermore, pre-mortality effects are known to occur following direct exposure to entomopathogenic fungi [14,15,16,17,18]; possible pre-mortality effects on reproductive capacity (i.e., per capita aphid fecundity) following exposure to endophytic entomopathogenic fungi have yet to be investigated. 

Many behavioral responses in insects (foraging, finding mates and oviposition sites, and avoiding natural enemies) are mediated by olfactory chemical cues emitted by both the plants and the insects [19]. Plant volatile emissions can also change when the plant is colonized by microorganisms, which can influence the responses of herbivorous insects and their natural enemies to those plants [20,21,22,23,24,25]; whether endophytic colonization induces analogous effects remains unknown, even though it has been reported that entomopathogenic fungi do emit volatiles and that microbial volatiles may modify insect interactions [26,27]. In this study, we determined the lethal and pre-mortality effects of several isolates of entomopathogenic fungi against the cotton aphid *Aphis gossypii* Glover when the aphids were either exposed to leaves that were both sprayed with the fungal suspension and endophytically colonized or only endophytically colonized by the fungi. Aphid preference for uncolonized or endophytically-colonized melon plants was evaluated, as well as the volatile emissions from endophytically-colonized and uncolonized plants. 

## 2. Materials and Methods

### 2.1. Biological Material: Aphid Colonies, Plants and Fungal Isolates

A virus-free laboratory population of *A. gossypii* was provided by the Institute of Agricultural Sciences (ICA) CSIC (Madrid, Spain) for use in the assays. The aphids were then maintained in rearing cages on melon plants (*Cucumis melo* L. cv. Siglo) for several generations in an environmental growth chamber under controlled conditions: 25 ± 2 °C, 16:8 h light:dark regime, and 70% RH. For each experiment, newly emerged apterous adult females (24–72 h after last moult) were collected from the rearing cages using a camel-hair brush and used immediately in experiments. 

Certified endophyte-free melon seeds were surface sterilized in 2% NaOCl (Sigma-Aldrich, MO, USA) for 2 min, rinsed twice with sterile Mili-Q water and dried under a sterile airflow. The soil substrate in which they were to be grown was sterilized in an autoclave for 20 min at 121 °C; this was done twice with a 24 h interval between each sterilization. Seeds were germinated in 9x9 cm pots using a mixture of equal parts of vermiculite (No. 3, Asfaltex S.A., Barcelona, Spain) and sterilized soil substrate (Floragard, Oldenburg, Germany) and maintained in an environmental chamber under controlled conditions: 25 ± 2 °C, 16:8 h light:dark regime. Plants were watered three times a week and a nutrient complex of 20-20-20 (N:P:K) Nutrichem 60 fertilizer (Miller Chemical & Fertilizer Corp., Hanover, PA, USA) was added to the irrigation water in a proportion of 1 g/L. 

Two isolates of *Beauveria bassiana* and one isolate of *Metarhizium brunneum* were used in the assays; all isolates had verified endophytic activity when inoculated onto melon plants [9,10]. These isolates are deposited at the University of Córdoba Entomopathogenic Fungi Collection, Córdoba, Spain and full details can be found in Table 1. All isolates were grown over cellophane film in Petri dishes of Potato Dextrose Agar (PDA) (Becton Dickinson Franklin Lakes, NJ, USA); the cellophane between the agar and the fungus was used to prevent nutrients entering the conidial suspensions at harvest. Cultures were incubated for 15 days at 25 °C in darkness and conidial suspensions were prepared by scraping the fungus from the cellophane into a sterile aqueous solution of 0.01% Tween 80. The resulting suspension was filtered through several layers of sterile cheesecloth to remove mycelia, and sonicated for 5 min to homogenize the inoculum. Concentration of viable conidia used for inoculation was determined using a haemocytometer and appropriate dilutions were made in 0.01% Tween 80 to obtain a conidial concentration of 10^8^ conidia/ml. Prior to experimentation, conidial viability was determined on liquid Czapek-Dox broth plus 1% (w/v) yeast extract medium and only suspensions with >97.0% germination after 24 hours were used.

### 2.2. Inoculation of Melon Plants with Entomopathogenic Fungi and Verification of Endophytic Colonization

For each experiment and each fungal isolate, replicate groups of four-leaf-stage melon plants were treated. Two leaves per plant were sprayed with 2 mL of the fungal suspension with an aerograph 27085 (piston compressor of 23 L/min, 15–50 PSI and a 0.3 mm nozzle diameter, Artesanía Latina S.A., Lamadrid, Spain). One millilitre of 10^8^-conidial suspension was sprayed over each leaf, resulting in a spray deposition at the level of the target surface of approximately 0.1 µL/mm^2^. Using the CFU method, we estimated that the suspension produced a deposition of 10,000 viable conidia/mm^2^.

During application, the remaining two leaves per plant were protected from the spray with a transparent plastic sheet and remained uninoculated. After inoculation, all plants were covered by another plastic sheet to promote fungal growth for 24 h. Control plants were treated in the same way but only sprayed with sterile water with 0.01% Tween 80. 

To confirm endophytic colonization, leaves were collected when each experiment had finished to avoid damaging the plant and triggering plant defenses which may have confounded the results. Inoculated and uninoculated leaves were sampled from each replicate plant, surface-sterilized with 1% NaOCl for 2 min, rinsed twice in sterile distilled water, and dried on sterile filter paper. Sections of approximately 2 cm^2^ were cut with a sterile scalpel from each leaf and plated out independently in Petri dishes containing selective culture medium to determine the percentage colonized endophytically; the medium contained: 20 g of Agar Sabouraud Glucose Chloramphenicol (Cultimed, Panreac Química S.A., Barcelona, Spain), 500 mg/L streptomycin sulfate (Sigma-Aldrich Chemie, Steinheim, Germany), 500 mg/L ampicillin (Intron biotechnology DR, Seongnam, Korea) and 500 mg/L dodine 65 WP (BASF Española S.L., Barcelona, Spain). We also plated out the final rinse water from each leaf separately to confirm the effectiveness of the surface-sterilization procedure. All plates were incubated at 25 ˚C in darkness until fungal growth was observed. Only data obtained from confirmed endophytically colonized leaves were used in the results.

### 2.3. Effect of Endophytic Plant Colonization by Entomopathogenic Fungi on Aphid Mortality and Fecundity 

To assess mortality and pre-mortality effects of the fungal isolates on aphids five melon plants per isolate were sprayed as described above. Forty-eight hours after spraying, five apterous adult aphids (7 days old) were transferred, using a camel-hair brush, to sprayed and unsprayed leaves in treatment and control melon plants respectively. Each group of five aphids was confined in a clip cage and mortality and fecundity were recorded every two days for one week. On each occasion, newly deposited nymphs were removed from each clip cage once they had been counted. When dead adult aphids were observed they were removed from the clip cage and immediately surface sterilized in 1% NaOCl for 30 s, followed by two rinses with sterile water. Cadavers were placed on sterile wet filter paper in sterile Petri plates sealed with Parafilm^®^ and incubated at 25 °C in darkness until fungal growth on the surface of the insect cuticle was observed. The entire experiment was repeated using new plants, aphid specimens and fungal inoculum.

### 2.4. Effect of Entomopathogenic Fungal Plant Colonization on Aphid Behavior

**Figure 1:**
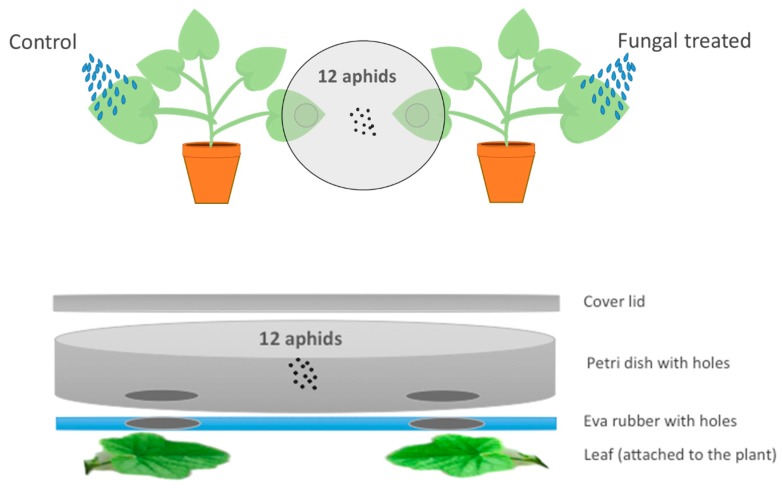
Design of an experimental arena for the choice bioassay. The set-up consists of one large Petri dish (150 mm × 15 mm) with two holes (diameter 25 mm each) in the bottom and covered by the lid. The area in contact with the plant was lined with eva rubber to avoid damaging the leaves. Aphids could choose between an unsprayed but endophytically-colonized leaf and a leaf from the control plant, or they could remain in the neutral area (no choice). The final position (endophytically-colonized leaf, control leaf, or neutral area) was recorded.

Treated and control plants were produced as described previously. Aphid behavior was assessed in an assay where, for each fungal isolate, aphids were offered a choice between an endophytically-colonized leaf and an untreated control leaf; both leaves remained attached to the plant (Figure 1). One unsprayed leaf from the fungus-inoculated plant (endophytically-colonized) and one from the control plant were placed under each of two holes (diameter 25 mm each) made in the bottom of a Petri dish (150 mm × 15 mm) and covered by the lid. The area in contact with the plant was lined with eva rubber to avoid damaging the leaves. This created an arena where the leaves were positioned 7 cm apart from each other and aphids had access to 5 cm^2^ from each plant. The positions of endophytically-colonized and untreated leaves in the Petri dish (left or right) were randomized to avoid any positional bias during observations. Twelve 24-hour starved aphids were introduced into the middle of the Petri dish helped by a paintbrush and incubated at 25 ± 2 ˚C, 16:8 h light:dark regime, and 70% RH. After 24 h their position (on the endophytically-colonized leaf, on the control leaf or on neither leaf) was recorded. There were three replicate arenas for each fungal isolate. The experiment was repeated using new plants, aphid specimens and fungal inoculum each time.

### 2.5. Analysis of Volatile Compounds from Aphid-Infested, Endophytically-Colonized Leaves 

Treated and control plants were produced as described previously. Five adult aphids were retained in clip cages on one sprayed and one unsprayed leaf from each replicate treated and control plant and incubated for 6 days at 25 ± 2 ˚C, 16:8 h light:dark regime, and 70% RH. There were 18 replicate treated plants per isolate and 18 control plants. After 6 days, the leaves on which the aphids had been feeding were sampled and the volatiles evaluated. Homogenates of each leaf were prepared according to Sánchez-Ortiz et al. [28]. Briefly, 3 g from each leaf was homogenized for 1 min at 19,500 rpm in 6 mL of distilled water using an A 11 IKA analysis mill (IKA, Werke GmbH & Co.KG, Staufen, Germany). The sample was allowed to equilibrate for 5 min at 25 °C; 1 mL aliquots of homogenate were placed in vials containing the same volume of a saturated CaCl_2_ solution, sealed and stored at −20 °C prior to analysis.

Volatile compounds were extracted from the samples using solid-phase microextraction (SPME) and analyzed using a High-Resolution Gas Chromatograph with Triple Quadrupole systems Mass Spectrometry (HR-GC-TQ MS) (Bruker model Scion 456-GC-TQ MS system; Bruker, MA, USA). Method parameters were set based on previous work [28]. Briefly, each sample was heated to 40 °C and allowed to equilibrate for 10 min; a preconditioned SPME fiber (50/30 μm DVB/CAR/PDMS Stableflex 23Ga, Autosampler, SUPELCO, Bellefonte, PA, USA) was then introduced into the vial and volatile compounds were adsorbed for 50 min. Desorption of volatile compounds was done directly into the GC injector at 250 °C for 5 min. A Supelcowax 10 capillary column (30 m × 0.25 mm, 0.25 μm, Sigma-Aldrich Co. LLC, St. Louis, MO, USA) was used with the following parameters: Helium as the carrier gas; injector and detector set at 250 °C; column held for 5 min at 40 °C and then the temperature programed to increase at a rate of 4 °C min^−1^ until it reached 200 °C. 

Identification of the volatile compounds detected was done by comparing their retention times against available standards purchased from Sigma-Aldrich-Merck, matching them to the NIST Library, or by comparing their MS spectra with reference spectra from different libraries (resemblance percentage above 80%). The method used for the identification was as follows: EI mode (70 eV), ion source and transfer line temperatures were all fixed at 250 °C. Mass spectra were obtained in full scan mode in the 300 to 400 mass-to-charge ratio range at a scanning speed of 7 scan/s. Chromatograms and spectra were recorded and processed using the Bruker MS Workstation version 8.2 (Bruker, Billerica, MA, USA). Volatile compounds were organized according to their chemical structure with respect to aldehydes, alcohols, ketones, terpenoid and phenolic derivatives and they were semi-quantified using an external standard calibration for each chemical group using hexanal, 1-octen-3-ol, 6-methyl-5-hepten-2-one, farnesene and ethylbenzene as standards, respectively. Each compound was semi-quantified against the external standard calibration of the corresponding chemical group in mg/L of homogenate and analyzed three times in duplicate experiments.

### 2.6. Statistical Analysis

Aphid mortality (%) was analyzed using the generalized linear mixed model method, with logit link and binomial distribution$:{\;\mathrm{Mortality}}_{ijk}=\mu +treatment_{i}+experiment_{j}$, where *k* indicates plant (replicate). Treatment was modelled as a fixed effect and experiment was modelled as a random effect. The estimation method was maximum likelihood with Laplace approximation. Significance of the fixed effect (Treatment) was evaluated using the F-approximate test (α = 0.05) and means from the different treatments were compared to the control using the Dunnett’s test (α = 0.05). Mortality data obtained from these experiments were also subjected to Kaplan–Meier survival analysis [29] to calculate Average Survival Time (AST) values in days and compared by the Log-rank test.

The number of nymphs produced each day per aphid was analyzed using a linear mixed model for repeated measures to evaluate main effects and interactions between treatment and day:

(2) $$No.\;of\;nymphs\;per\;aphid_{ijk}=\mu +treatment_{i}+day_{j}+treatment\times day_{ij}$$

Correlation between repeated measures was modelled as an autoregressive covariance matrix. Each replicate (plant), denoted by *k*, was the experimental unit for repeated measures. Treatment and day were modelled as fixed effects. The factor experiment was initially included in the model as a random effect, but it was not significant and, therefore, was removed from the model and the two experiments were combined in the same analysis. The linear mixed model was estimated using the restricted maximum likelihood (REML) method, and means were compared using the Dunnett’s test (α = 0.05) [30].

Aphid choice (between endophytically-colonized and control plants) was analyzed using a Pearson Chi-square test (*p* ≤ 0.05) to determine whether the observed frequencies were significantly different to the expected ones (50% : 50%). Data on volatile compounds were analyzed using Shapiro–Wilk’s and Levene’s tests to evaluate linear model assumptions (normality and homogeneity of variance) and analysis of variance (ANOVA) applied at a significance level of 0.05. Mixed models were analyzed with proc GLIMMIX-SAS 9.3 [31]; Pearson Chi-square, Kaplan Meier and linear models were analyzed with SPSS 24.0 [32].

## 3. Results

### 3.1. Endophytic Colonization of Melon Plants

Microbiological techniques confirmed endophytic fungal colonization in 100% of the leaves that had received fungal sprays directly, but in only 20–40% of the leaf sections from unsprayed leaves from treated plants. Individual plants were considered as positive for endophytic colonization based on detection of fungus within any of the leaves sampled on that plant. 

### 3.2. Effect of Endophytic Colonization on Aphid Mortality 

The likelihood ratio tests to evaluate the effect of the experiment for both fungus sprayed leaves and unsprayed leaves were not significant (χ2 _1;_ *p* > 0.05). For leaves sprayed with the fungal suspension (i.e., leaves with conidia on the surface and endophytically colonized), there was, overall, a significant effect of treatment on aphid mortality (F _3,28_ = 5.63; *p* = 0.0038; Table 2); there were significant differences between isolates and the control according to the Dunnett’s test (EABb 01/33-Su: t_28_ = 3.31; *p* = 0.006; EABb 04/01-Tip: t_28_ = 3.81; *p* = 0.0017; EAMa 01/58-Su: t_28_ = 3.60; *p* = 0.0029) (Table 2). For leaves that were only endophytically colonized (i.e., leaves not sprayed with the fungal suspension but on the same plant as fungus-sprayed leaves) the overall effect of treatment on mortality was also significant (F_3,28_=3.36; *p* = 0.0328; Table 3). The mortality caused by isolate EABb 01/33-Su was significantly higher than the mortality in the control (Dunnett’s test t_28_ = 3.01, *p* = 0.0135; Table 3). There were no significant differences between the mortalities caused by isolates EABb 04/01-Tip and EAMa 01/58-Su and the mortality in the control at α=0.05, although these differences were statistically significant at α = 0.1 (Dunnett’s test t_28_ = 2.22, *p* = 0.0796 for strain EABb 04/01-Tip; Dunnett’s test t_28_ = 2.36, *p* = 0.0585 for strain EAMa 01/58-Su; Table 3).

### 3.3. Effect of Endophytic Colonization of Plants on Aphid Fecundity

**Figure 2:**
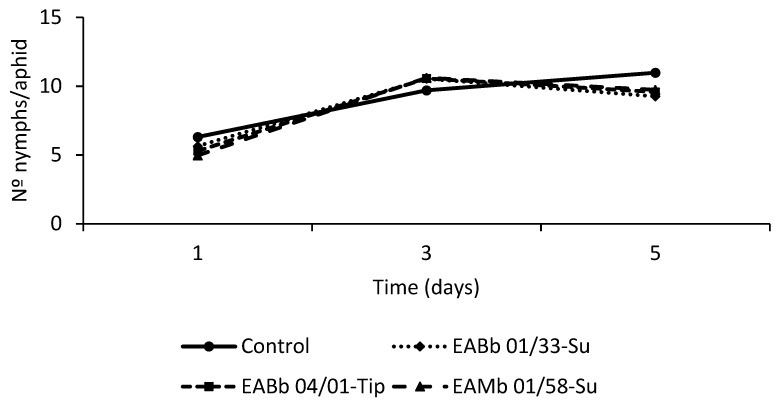
*Per capita* aphid fecundity over time (5 days) after aphid exposure to a sprayed leaf (with both surface conidia and endophytic colonization by one of three fungal isolates or the control). Evaluation began (day 1 on graph) 72 hours after leaves had been sprayed. Data represents the average of two trials.

**Figure 3:**
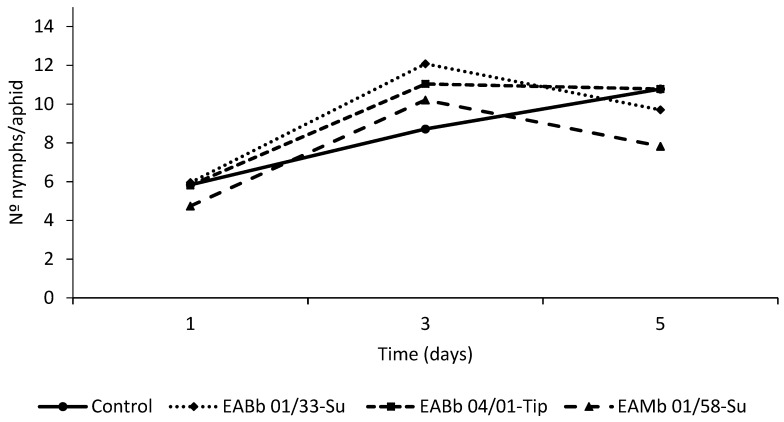
*Per capita* aphid fecundity over time (5 days) after exposure to an unsprayed leaf (endophytically-colonized by one of three fungal isolates or the control). Evaluation began (day 1 on graph) 72 hours after leaves had been sprayed. Data represents the average of two trials.

The likelihood ratio tests to evaluate the effect of the experiment for both fungus sprayed leaves and unsprayed leaves were not significant (χ^2^_1;_ *p* > 0.05) and therefore the two experiments were combined. The analysis of the pooled fecundity data indicated that there was a significant effect of time on the number of nymphs deposited by aphids feeding both on fungus-sprayed leaves and unsprayed but endophytically-colonized leaves (F_2,69.8_ = 52.01; *p* < 0.001 and F_2,69.8_ = 44.25; *p* < 0.001 respectively). However, there was no significant effect of treatment (F _3,55.07_ = 0.29; *p* = 0.84) or a treatment*day interaction for the fungus-sprayed leaf (F _6,84.2_ = 0.82; *p* = 0.56) (Figure 2). Conversely, for the unsprayed but endophytically-colonized leaf, the effect of treatment and the treatment*day interaction were significant, at α = 0.1 (treatment: F_3,56.6_ = 2.35; *p* = 0.08; treatment*day interaction: F_6,81.7_= 2.03; *p* = 0.07) (Figure 3). The maximum reproductive activity of aphids was attained earlier in aphids feeding on endophytically-colonized leaves than in the controls (Figure 3). In fact, significant differences were detected by day 3 of the assay, if the number of nymphs laid by aphids feeding on leaves endophytically-colonized by isolate EABb 01/33-Su is compared with the number laid by aphids feeding on control leaves (t _103_ = 2.89; *p* = 0.01).

**Figure 4:**
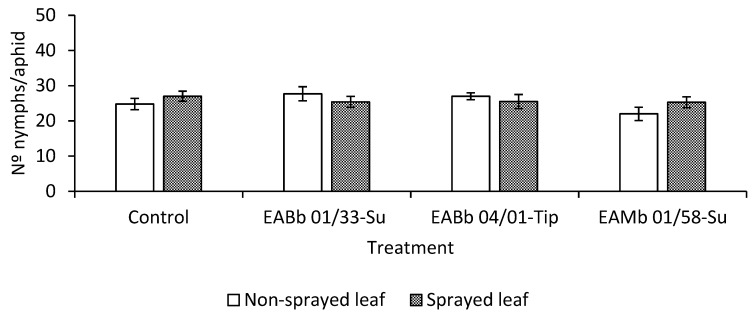
Total *per capita* aphid fecundity 5 days after applying the aphids to the plant (7 days after leaves had been sprayed). Fecundity data are pooled from the two occasions that the experiment was run.

Nonetheless, although fecundity was accelerated, this did not result in an overall increase in the aphid population compared with the control (Figure 4). At the end of the assay (day 5), aphids feeding on endophytically-colonized leaves laid, in general, fewer nymphs than ones feeding on control plants. This was particularly apparent in aphids feeding on leaves endophytically-colonized by isolate EAMa 01/58-Su (t _103_ = −2.49; *p* = 0.04).

### 3.4. Effect of Endophytic-Colonization of Plants on Aphid Behavior

**Figure 5:**
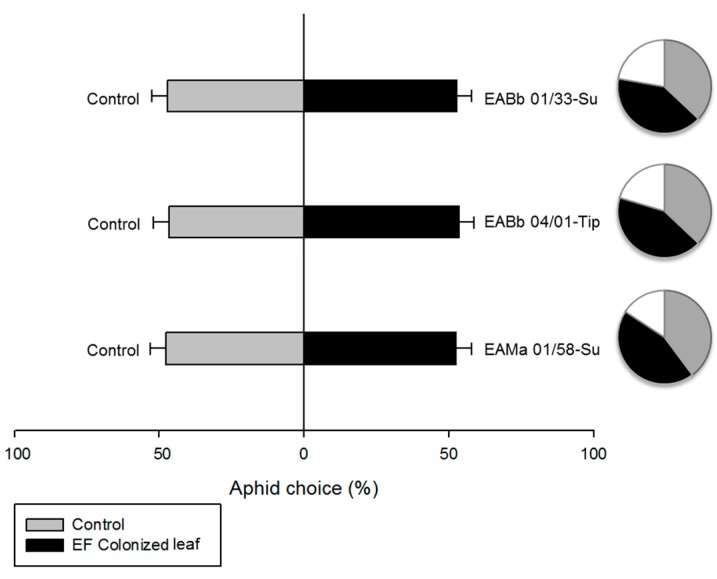
Percentage of *Aphis gossypii* adults choosing one of the two leaves offered in the choice experiment. The following choices were offered: Unsprayed leaf from a fungus-sprayed plant (i.e., endophytically colonized only); Unsprayed leaf from a control plant. Bars denote percentage of aphids choosing either the control or the endophytically-colonized leaf out of the total number of aphids making a choice. Circles denote the proportion of aphids orienting towards the unsprayed endophytically-colonized leaf (black), the unsprayed leaf from a control plant (grey) and aphids showing no decision (white).

Endophytic colonization of leaves by entomopathogenic fungi did not influence host plant selection by aphids (Figure 5). There were no significant differences in the number of aphids on control and endophytically-colonized leaves when the aphids were offered a choice between the two, and this was consistent for all isolates either in the analyses by experiment (χ^2^_1;_ *p* > 0.05) or in the analyses with the experiments combined: EABb 01/33-Su (likelihood ratio test χ2 _1_ = 0.19; *p* = 0.66); EABb 04/01-Tip (likelihood ratio test χ2 _1_ = 0.41; *p* = 0.52); EAMb 01/58-Su (likelihood ratio test χ2 _1_ = 0.27; *p* = 0.60).

### 3.5. Volatile Compounds from Aphid-Infested and Endophytically-Colonized Leaves 

**Figure 6:**
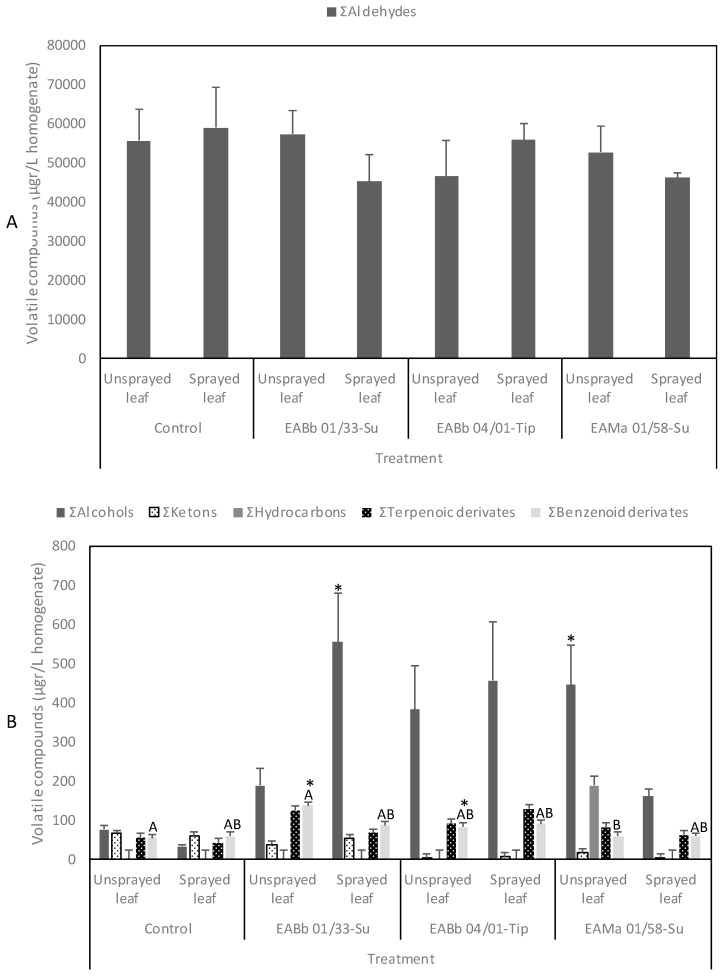
Aldehydes (**A**) and alcohols, ketones, hydrocarbons, terpenoid and phenolic derivatives (**B**) (Mean ± SE, from the two trials) emitted by melon leaves from different treatments (unsprayed leaves and sprayed leaves) in control and inoculated plants by different isolates: EABb 01/33-Su, EABb 04/01-Tip and EAMa 01/58-Su. Statistically significant differences were determined using the LSD (α = 0.05).

A large number of volatile organic compounds (VOCs) were emitted from aphid-infested, endophytically-colonized leaves: terpenoids, phenylpropanoids/benzenoids, amino acid derivatives and fatty acid derivatives such as: aldehydes, alcohols, and ketones (Figure 6). Aldehydes were the main group of VOCs emitted by plants; E-2-hexenal and (E,Z)-2,6-nonadienal were emitted at the highest concentrations. In contrast, terpenoid and phenolic derivatives were emitted in the lowest concentrations. The full listing of VOCs emitted are in Supplementary Tables S1–S3.

These VOCs are biosynthesized as primary and secondary metabolites by complex enzyme pathways distributed across different organelles or compartments within the plant. In general, the vegetative tissues of melon only release small quantities of VOCs but can be induced by mechanical damage by herbivores. In this regard, the use of homogenized melon plants (i.e., complete destruction of plant tissue) was effective in establishing differences in the volatile profiles emitted from leaves that had been inoculated with different isolates of fungi compared with controls. 

Finally, from Supplementary Tables S1–S3 VOCs could largely be grouped into Σaldehydes, Σalcohols, and Σketones. Specifically: Σaldehydes = butanal, 3-methyl-butanal, pentanal, hexanal, 3-methyl-hexanal, (E)-2-pentenal, heptanal, 5-methyl-hexanal, (Z)-2-hexenal, (E)-2-hexenal, (Z)-4-heptenal, octanal, (Z)-2-heptenal, (E)-2-octenal, (E)-6-nonenal, (E,E)-2,4-hexadienal, nonanal, (E,E)-2,4-heptadienal, (E)-2-nonenal, (E,Z)-2,6-nonadienal, (E)-4-oxohex-2-enal; Σalcohols = ethanol, 1-penten-3-ol, 4-methyl-1-pentanol, (Z)-3-hexen-1-ol, (E)-2-hexen-1-ol, 1-octen-3-ol, 2-ethyl-1-hexanol, 2-propyl-1-pentanol; Σketones = 6-methyl-2-heptanone, 3-octanone, 6-methyl-5-hepten-2-one, (E,E)-3,5-octadien-2-one. There were also: terpenoic derivates = β-cyclocitral, eucarvone, 2-pinen-4-ol, β-citral, geranial, isomethyl-α-ionone, E-β-ionone, 2,2,6-trimethyl-cyclohexanone, β-ionone; and Σphenolic derivates = benzaldehyde, acetophenone, benzyl alcohol, 2-phenyl-isopranol, phenol, benzophenone, toluene. 

## 4. Discussion

This study emphasizes the benefits of entomopathogenic fungi for crop protection that are in addition to the direct mortality that the fungus causes. Aphids may come into contact with conidia on the surface of sprayed leaves that are also endophytically colonized, or with leaves on the same plant that have not been sprayed so have no surface conidia, but are endophytically colonized. 

Using microbiological techniques, high levels of endophytic fungal colonization were detected within fungus-sprayed leaves, while endophytic fungi were recovered from 20–40% of leaf sections from unsprayed leaves on the same plants as the sprayed leaves. The use of more sensitive endophytic colonization-detection techniques may allow us to quantify the extent of colonization more accurately and decrease the risk of false negatives. 

Despite this, our results showed a significant decline in aphid survival on fungus-sprayed leaves that were also endophytically-colonized (leaves sprayed with the fungal suspension) compared with controls and this was consistent for all fungal isolates evaluated. However, when aphids fed on leaves that were only endophytically colonized there was only a significant difference in aphid mortality on leaves colonized with isolate EABb 01/33-Su compared with the control. Nevertheless, aphid mortality on leaves colonized by isolates EABb 04/01-Tip and EAMa 01/58-Su was significantly greater that the control at the 10% significance level. These results are consistent with the study of Garrido-Jurado et al. [10], who also demonstrated that isolate EABb 01/33-Su had greater potential to control sap-sucking insects as an endophyte compared with the other isolates.

A reduction in aphid reproduction on endophytically-colonized plants has been demonstrated previously [16,17,18,33]. In our study we did not observe any effect of endophytic colonization on total *per capita* aphid fecundity 6 days after exposure (8 days after leaf spraying). However, we did show, for the first time, a modification in the pattern of aphid fecundity during feeding on uninoculated but endophytically-colonized leaves; endophytic colonization accelerated aphid reproductive activity, revealing a different reproductive strategy than their control counterparts. This response ensured reproductive success achieved its full potential before the onset of fungal infection and death [34].

We observed significant qualitative and quantitative differences in the blend of volatiles released by endophytically-colonized and control leaves, which have potential to influence insect behavior. This is the first attempt to describe the total blend of volatiles released by endophytically-colonized melon leaves following herbivore damage. These VOCs are biosynthesized as primary and secondary metabolites by complex enzyme pathways distributed across different organelles or compartments within the plant. In general, the vegetative tissues of melon only release small quantities of VOCs but can be induced by mechanical damage by herbivores. In this regard, the use of homogenized melon plants (i.e., complete destruction of plant tissue) was effective in establishing differences in the volatile profiles emitted from leaves that had been inoculated with different isolates of fungi compared with controls. 

When endophytically-colonized and control melon leaves were infested by *A. gossypii*, our volatile analysis revealed the presence in both treatments of a characteristic blend of melon plant volatiles that has been described previously [35]; this blend is derived from five biosynthetic classes: aldehydes, alcohols, ketones, terpenoids and phenolic compounds. A number of the compounds we recorded here were found in previous publications on melon volatiles (compounds 4, 7, 9, 17 25, 32, and 39 in Table S1, Supplementary Material) [35]. Furthermore, many volatile compounds we found have been reported in a wide range of plant species and are regarded as herbivore-induced plant volatiles (compounds 4, 9, 25, and 42 in Table S1, Supplementary material) [36,37,38]. For example, the release of benzyl alcohol has been reported in *Camellia sinensis* and *Coffea canephora* under attack from different insect-feeding guilds [39] and even when mechanically damaged [40]. In aphid species, perception of some of these plant-specific volatile component assists olfactory discrimination between host and non-host plants [41].

Some of the compounds we identified were only detected in endophytically-colonized plants (compounds 8, 24, 29, 34, 38, 40, 41, 43, 46, and 47 Table S1, Supplementary Material) or were only detected in control plants and suppressed in endophytically-colonized plants (compounds 2, 3, 5, 10, 12, 13, 22, 27, 29, 35, 36, 38, 41, and 47 Table S1, Supplementary Material). These differences in production of particular VOCs could be as a result of endophytic colonization by entomopathogenic fungi. Many different alkyl benzenes have been retrieved previously from plants inoculated with entomopathogenic fungi and infested by *Delia radicum* (Diptera: Anthomyiidae), especially in the presence of high densities of entomopathogenic fungal inoculum [42]. For example, we only detected benzyl alcohol in endophytically-colonized plants. This compound, together with methyl benzoate and benzaldehyde, are known to elicit very strong responses in the important pollinators *Manduca sexta* and *Sphinx perelegans* (Lepidoptera: Sphingidae) [43,44]. Benzaldehyde, which was found in high levels in leaves that had been sprayed with isolate EABb 04/01-Tip, is thought to be a biomarker for pathogenic infections in algae, together with 1-octen-3-one and E-2-nonenal [45].

We detected the terpenoids and carotenoid derivatives, beta-ionone and trans-beta-Ionone only in endophytically-colonized plants. It is reported that carotenoid derivatives affect insect behavior [46]. For example, beta-ionone is highly attractive to beetles including *Anomala transvaalensis* (Coleoptera: Scarabaeidae) [47]. However, this compound is repellent to *Cnaphalocrocis medinalis* (Lepidoptera: Pyralidae) [48]. Beta-ionone is also present in rice extracts and attracts rice planthoppers [49]. 

Another fact that is immediately apparent from this study is the great increase in alcoholic compounds released by fungal sprayed and endophytically-colonized leaves (Figure 6), although there was no difference in the quantity of aldehydes, ketones, terpenoids and phenolic compounds when they were considered as a group.

In the present study, endophytic colonization of leaves by entomopathogenic fungi did not influence host plant selection by aphids, which is likely to be the case for most insect-microbe associations [26]. There have been a few studies evaluating the effect of endophytic colonization by entomopathogenic fungi on host plant selection behavior by herbivores [50,51,52]. In some cases, phytophagous insects are able to detect and subsequently avoid plants treated with entomopathogenic fungi [51,52,53] while others are attracted by plants treated with entomopathogenic fungi [50]. A major difference between these experiments and our own was the phenological stage of the plant and how long the plant had been colonized by the entomopathogenic fungi, which was longer than in our study. It might be expected that, in older plants, the volatiles emitted would be different and could change the results. Furthermore, herbivore behavior was measured for 6 hours, and the deterrent effect was more evident at the beginning of the experiment than at the end, when final establishment of the herbivore was recorded. Results may have been different if a shorter response time had been used.

## 5. Conclusions 

The results obtained in this study support the hypothesis that following a spray application of entomopathogenic fungi, plant defense continues beyond the initial pest mortality caused, as a result of the endophytic capacity of some isolates. However, the effects described should now be confirmed under greenhouse and field conditions.

Further research on the volatile compounds released by plants entomopathogenic fungi endophytically colonizing would lead to a deeper understanding of the behavioral responses of the insect community associated with these plants. The benefits of endophytic colonization by entomopathogenic fungi for sustainable crop protection could be exploited more fully; these include direct and indirect defense, pathogen resistance and abiotic stress tolerance.

## Figures and Tables

**Table 1:** Origin of the fungal isolates used in the assays.

Isolate	Fungal Species	Origin	Agroecosystem	Habitat	GenBank Accession Number	Spanish Type Culture Collection (CECT ) Accession Number
EABb 04/01-Tip	*B. bassiana*	Ecija (Sevilla, Spain)	Opium poppy crop	Insect (*Iraella luteipes*)	FJ972963	20744
EABb 01/33-Su	*B. bassiana*	El Bosque (Cádiz, Spain)	Traditional olive Orchard	Soil	FJ972969	-
EAMa 01/58–Su	*M. brunneum*	Hinojosa del Duque (Córdoba, Spain)	Wheat crop	Soil	JN900390	20764

**Table 2:** Susceptibility of *A. gossypii* adults to entomopathogenic fungal isolates after exposure to leaves with conidia on their surfaces and which were also endophytically-colonized by those fungi.

Treatment	Mortality (%)	Kaplan-Meier Survival Analysis		
	Mean (± SE) *^a^*	AST *^b^* (± SE; Days)	Confidence Interval (95%)	
			Lower Limit	Upper Limit
Control	0.80 ± 0.06	6.56 ± 0.25 a	6.07	7.05
EABb 01/33-Su	48.15 ± 0.18 *	6.15 ± 0.28 b	5.60	6.71
EABb 04/01-Tip	56.92 ± 0.18 *	6.18 ± 0.25 b	5.70	6.66
EAMa 01/58-Su	53.68 ± 0.18 *	5.77 ± 0.28 b	5.22	6.33

*^a^* Means with * are significantly different from the control according to the Dunnett’s test (α = 0.05). *^b^* AST: Average survival time. Means within columns with the same lower-case letter are not significantly different to each other according to the log rank test (*p* < 0.05). Average survival time was limited at 7 days. Data are pooled from the two occasions on which the experiment was run.

**Table 3:** Susceptibility of *A. gossypii* adults to entomopathogenic fungal isolates after exposure to leaves that were endophytically-colonized by those fungi.

Treatment	Mortality (%)	Kaplan-Meier Survival Analysis		
	Mean (± SE) *^a^*	AST *^b^* (± SE; Days)	Confidence Interval (95%)	
			Lower Limit	Upper Limit
Control	13.74 ± 0.08	6.62 ± 0.21 a	6.22	7.03
EABb 01/33-Su	49.99 ± 14.09 *	5.90± 0.32 b	5.28	6.52
EABb 04/01-Tip	37.71 ± 13.61	6.41 ± 0.28 a	5.85	6.96
EAMa 01/58-Su	40.08 ± 13.56	6.11 ± 0.25 b	5.62	6.60

^a^ Means with * are significantly different from the control according to the Dunnett’s test (α = 0.05). *^b^* AST: Average survival time. Means within columns with the same lower-case letter are not significantly different to each other according to the log rank test (*p* < 0.05). Average survival time was limited at 7 days. Data were pooled from the two occasions on which the experiment was run.

## Supplementary Materials

The following are available online at https://www.mdpi.com/2075-4450/10/6/165/s1, Table S1: * Mean value from three measurements in two different experiments (μg/L homogenate). Values for each volatile with different letters in the same row within each treatment (control and EABb 01/33-Su isolate leaves) are significantly different to each other (*p* < 0.05)., Table S2: * Mean value from three measurements in two different experiments (μg/L homogenate). Values for each volatile with different letters in the same row within each treatment (control and EABb 04/01-Tip isolate leaves) are significantly different to each other (*p* < 0.05)., Table S3: * Mean value from three measurements in two different experiments (μg/L homogenate). Values for each volatile with different letters in the same row within each treatment (control and EAMa 01/58-Su isolate leaves) are significantly different to each other (*p* < 0.05).
