# Supplementary material for: Effects of Endophytic Entomopathogenic Ascomycetes on the Life-History Traits of Aphis gossypii Glover and Its Interactions with Melon Plants
Source: Insects. 2019 Jun 10;10(6):165. doi: 10.3390/insects10060165 (PMC6627330; doi:10.3390/insects10060165)
Supplement: Supplementary file 1 [file insects-10-00165-s001.pdf]

Table 1: *Mean value from three measurements in two different experiments (µg/L homogenate). Values for each volatile with different letters in the same row within each treatment (control and EABb 01/33-Su isolate leaves) are significantly different to each other ($P < 0.05$).

		Control				EABb 01/33-Su			
	Volatile compound*	Unprayed leaf		Sprayed leaf		Unsprayed leaf		Sprayed leaf	
	**Aldehydes**								
1	Butanal	33.46 ± 1.46		34.20 ± 1.27		32.72 ± 6.51			
2	3-methyl-Butanal	516.44 ± 64.81		460.33 ± 160.92		36.40 ± 16.99			
3	Pentanal	277.76 ± 51.60	A	100.53 ± 25.31	BC	132.58 ± 18.69	B		
4	Hexanal	2821.38 ± 490.76	A	2258.31 ± 587.30	A	2529.28 ± 358.45	AB	256.45 ± 84.93	B
5	3-Methyl-hexanal	29.94 ± 7.12		15.87 ± 2.86					
6	(E)-2-Pentenal	455.69 ± 59.25		603.18 ± 82.50		428.06 ± 86.53		493.13 ± 47.47	
7	Heptanal	42.99 ± 3.99		30.98 ± 7.79		54.63 ± 5.16			
8	5-Methyl-Hexanal								
9	(E)-2-Hexenal	1002.89 ± 154.60		1270.47 ± 218.95		976.03 ± 53.39		983.02 ± 197.96	
10	(Z)-2-Hexenal	40983.33 ± 5616.16		50015.83 ± 8148.49		41826.67 ± 2350.12		41781.88 ± 5975.75	
11	(Z)-4-Heptenal	34.68 ± 6.16	A	26.42 ± 5.31	A	42.08 ± 3.40	AB		
12	Octanal	68.40 ± 10.32	A	67.36 ± 11.83	A	54.92 ± 7.66	A		
13	(Z)-2-Heptenal	157.52 ± 31.36		31.96 ± 7.42		52.55 ± 14.04		28.87 ± 8.40	
14	(E)-2-Octenal	43.70 ± 3.23	AB	87.15 ± 9.23	A	88.85 ± 8.94	AB		
15	(E)-6-Nonenal	1266.36 ± 185.46	A	337.33 ± 42.74	BC	1734.36 ± 605.31	AB		
16	(E,E)-2,4-Hexadienal	421.24 ± 38.15	AB	585.81 ± 75.03	A	293.68 ± 22.73	B	587.13 ± 110.96	A
17	Nonanal	364.74 ± 78.17	A	309.59 ± 47.52	A	164.30 ± 17.51	B		
18	(E,E)-2,4-Heptadienal	1370.72 ± 198.33		1195.76 ± 176.80		1061.41 ± 77.27		955.62 ± 189.68	
19	(E)-2-Nonenal	294.45 ± 14.72		26.91 ± 3.61		740.13 ± 324.88		20.98 ± 3.47	
20	(E,Z)-2,6-Nonadienal	5396.60 ± 983.01		1520.04 ± 536.14		7068.75 ± 2008.75		144.49 ± 55.75	
21	(E)-4-Oxohex-2-enal	32.89 ± 5.73	A	41.30 ± 10.39	A	23.72 ± 1.56	B		
	**Alcohols**								
22	Ethanol								
23	1-Penten-3-ol	2.30 ± 0.57		3.35 ± 0.93					
24	4-Methyl-1-pentanol					36.51 ± 7.81	AB	151.41 ± 25.21	A
25	(Z)-3-Hexen-1-ol	4.23 ± 0.33	B	1.72 ± 0.41	B	9.28 ± 0.24	B	145.18 ± 33.15	A
26	(E)-2-Hexen-1-ol	12.76 ± 2.71	B			48.22 ± 16.91	B	204.22 ± 54.75	A
27	1-Octen-3-ol	41.15 ± 4.35	A	11.76 ± 1.03	BC	50.77 ± 8.20	A	11.01 ± 0.85	B
28	2-Ethyl-1-hexanol	15.91 ± 2.81	A	16.17 ± 1.20	A	18.56 ± 5.92	AB		
29	2-Propyl-1-pentanol					25.90 ± 6.03	AB	43.64 ± 9.30	A
	**Ketones**								
30	6-Methyl-2-heptanone	0.66 ± 0.02	A	0.46 ± 0.04	AB				
31	3-Octanone	2.07 ± 0.39	B	0.65 ± 0.13	B	8.31 ± 1.89	AB	17.80 ± 4.93	A
32	6-methyl-5-Hepten-2-one	1.31 ± 0.18	B	0.75 ± 0.20	B	23.09 ± 3.37	AB	31.97 ± 4.34	A
33	(E,E)-3,5-Octadien-2-one	61.86 ± 3.43		60.23 ± 7.04		5.39 ± 0.58		4.30 ± 0.16	
	**Hydrocarbons**								
34	3-ethyl-1,4-Hexadiene								
	**Terpenoic derivates**								
35	β-Cyclocitral	14.79 ± 1.89	B	16.37 ± 4.05	B	13.29 ± 1.35	AB	18.37 ± 3.78	A
36	Eucarvone	3.88 ± 1.04		5.49 ± 2.28					
37	2-Pinen-4-ol	1.77 ± 0.02		1.02 ± 0.15					
38	β-Citral					2.17 ± 0.60	AB	3.48 ± 0.44	A
39	Geranial	32.71 ± 0.94		17.89 ± 3.00		58.98 ± 5.33		26.76 ± 0.52	
40	Isomethyl-α-ionone					19.78 ± 1.44			
41	E-β-ionone					18.92 ± 1.57			
42	2,2,6-Trimethyl -cyclohexanone	3.54 ± 0.39		2.39 ± 0.32		2.62 ± 0.32		4.28 ± 1.02	
43	β-Ionone					9.82 ± 0.43	A	14.57 ± 1.77	B
	**Phenolic derivates**								
44	Benzaldehyde	27.00 ± 3.74	AB	32.57 ± 3.93	AB	47.44 ± 13.63	A	21.21 ± 9.72	A
45	Acetophenone	7.28 ± 0.54		7.11 ± 0.45		7.59 ± 0.85		7.02 ± 0.72	
46	Benzyl alcohol					16.85 ± 4.67	B	31.53 ± 4.05	A
47	2-Phenyl-isopranol					19.93 ± 7.85		±	
48	Phenol	4.86 ± 0.54		3.82 ± 0.61		17.56 ± 4.43		9.77 ± 1.99	
49	Benzophenone	10.48 ± 0.91		10.36 ± 0.88		11.42 ± 1.39		10.23 ± 1.49	
50	Toluene	5.62 ± 0.19	B	6.08 ± 0.57	B	17.03 ± 3.50	A	8.16 ± 0.71	B

Table 2: *Mean value from three measurements in two different experiments (µg/L homogenate). Values for each volatile with different letters in the same row within each treatment (control and EABb 04/01-Tip isolate leaves) are significantly different to each other (*P* < 0.05).

	Control				EABb 04/01-Tip			
Volatile compound*	UnSprayed leaf		Sprayed leaf		Unsprayed leaf		Sprayed leaf	
**Aldehydes**								
Butanal	33.46 ± 1.46		34.20 ± 1.27		11.33 ± 0.87		33.23 ± 1.89	
3-methyl-Butanal	516.44 ± 64.81		460.33 ± 160.92		301.46 ± 26.42		167.43 ± 47.06	
Pentanal	277.76 ± 51.60	A	100.53 ± 25.31	B	116.04 ± 10.67	B	135.82 ± 9.21	B
Hexanal	2821.38 ± 490.76		2258.31 ± 587.30		1695.53 ± 291.95		2186.75 ± 386.69	
3-Methyl-hexanal	29.94 ± 7.12		15.87 ± 2.86		15.92 ± 7.34		6.66 ± 1.52	
(E)-2-Pentenal	455.69 ± 59.25	AB	603.18 ± 82.50	A	288.91 ± 66.72	B	469.59 ± 108.89	AB
Heptanal	42.99 ± 3.99	B	30.98 ± 7.79	B	180.43 ± 8.61	A		B
5-Methyl-Hexanal					180.73 ± 8.31			
(E)-2-Hexenal	1002.89 ± 154.60		1270.47 ± 218.95		670.75 ± 16.23		1185.10 ± 77.78	
(Z)-2-Hexenal	40983.33 ± 5616.16	AB	50015.83 ± 8148.49	A	36584.38 ± 8388.05	B	46529.17 ± 2377.70	A
(Z)-4-Heptenal	34.68 ± 6.16		26.42 ± 5.31		34.24 ± 0.49		40.58 ± 4.50	
Octanal	68.40 ± 10.32		67.36 ± 11.83		47.66 ± 7.54		46.78 ± 11.66	
(Z)-2-Heptenal	157.52 ± 31.36	A	31.96 ± 7.42	AB	20.35 ± 5.26	AB		B
(E)-2-Octenal	43.70 ± 3.23		87.15 ± 9.23		29.51 ± 1.02		26.39 ± 7.67	
(E)-6-Nonenal	1266.36 ± 185.46	A	337.33 ± 42.74	B	596.94 ± 82.56	B	432.87 ± 123.05	B
(E,E)-2,4-Hexadienal	421.24 ± 38.15		585.81 ± 75.03		303.41 ± 47.30		568.58 ± 107.23	
Nonanal	364.74 ± 78.17	A	309.59 ± 47.52	A	153.98 ± 4.42	B	136.14 ± 21.49	AB
(E,E)-2,4-Heptadienal	1370.72 ± 198.33	A	1195.76 ± 176.80	A	690.03 ± 96.92	B	1296.20 ± 224.05	A
(E)-2-Nonenal	294.45 ± 14.72		26.91 ± 3.61		377.94 ± 43.69		158.75 ± 60.25	
(E,Z)-2,6-Nonadienal	5396.60 ± 983.01		1520.04 ± 536.14		4198.75 ± 171.10		2312.73 ± 677.61	
(E)-4-Oxohex-2-enal	32.89 ± 5.73	AB	41.30 ± 10.39	A	9.05 ± 0.03	C	28.82 ± 8.19	BC
**Alcohols**								
Ethanol					279.00 ± 95.31		322.05 ± 121.65	
1-Penten-3-ol	2.30 ± 0.57		3.35 ± 0.93					
(Z)-3-Hexen-1-ol	4.23 ± 0.33		1.72 ± 0.41		15.23 ± 3.09		28.67 ± 12.41	
(E)-2-Hexen-1-ol	12.76 ± 2.71				14.83 ± 2.34		17.54 ± 0.58	
1-Octen-3-ol	41.15 ± 4.35	A	11.76 ± 1.03	C	35.57 ± 7.96	AB	40.80 ± 4.89	BC
2-Ethyl-1-hexanol	15.91 ± 2.81	A	16.17 ± 1.20	A	7.79 ± 2.26	B	20.14 ± 4.83	AB
2-Propyl-1-pentanol					30.34 ± 1.61		28.08 ± 5.39	
**Ketones**								
6-Methyl-2-heptanone	0.66 ± 0.02		0.46 ± 0.04					
3-Octanone	2.07 ± 0.39		0.65 ± 0.13		1.31 ± 0.32		4.13 ± 1.30	
6-methyl-5-Hepten-2-one	1.31 ± 0.18	A	0.75 ± 0.20	AB	0.70 ± 0.04	B	0.88 ± 0.24	B
(E,E)-3,5-Octadien-2-one	61.86 ± 3.43		60.23 ± 7.04		4.04 ± 0.84		3.28 ± 0.49	
**Hydrocarbons**								
3-ethyl-1,4-Hexadiene								
**Terpenoic derivates**								
β-Cyclocitral	14.79 ± 1.89		16.37 ± 4.05		12.65 ± 3.94		13.89 ± 2.64	
Eucarvone	3.88 ± 1.04		5.49 ± 2.28					
2-Pinen-4-ol	1.77 ± 0.02		1.02 ± 0.15					
β-Citral								
Geranial	32.71 ± 0.94		17.89 ± 3.00		15.27 ± 4.94		30.55 ± 7.57	
Isomethyl-α-ionone					7.33 ± 2.04		10.63 ± 3.43	
E-β-ionone					53.14 ± 1.43	AB	69.71 ± 3.75	A
2,2,6-Trimethyl-cyclohexanone	3.54 ± 0.39	A	2.39 ± 0.32	AB	1.37 ± 0.63	B	1.96 ± 0.92	AB
β-Ionone					1.86 ± 0.85	AB	2.92 ± 1.43	A
**Phenolic derivates**								
Benzaldehyde	27.00 ± 3.74		32.57 ± 3.93		32.92 ± 9.87		50.06 ± 15.58	
Acetophenone	7.28 ± 0.54		7.11 ± 0.45		6.77 ± 0.27		6.54 ± 0.55	
Benzyl alcohol					8.53 ± 1.27		10.38 ± 4.77	
2-Phenyl-isopranol					0.29 ± 0.02		0.28 ± 0.08	
Phenol	4.86 ± 0.54		3.82 ± 0.61		8.97 ± 2.56		6.69 ± 0.64	
Benzophenone	10.48 ± 0.91		10.36 ± 0.88		11.27 ± 0.89		12.62 ± 2.75	
Toluene	5.62 ± 0.19		6.08 ± 0.57		14.90 ± 9.91		5.36 ± 0.31	

Table 3: *Mean value from three mesurements in two different experiments (µg/L homogenate). Values for each volatile with different letters in the same row within each treatment (control and EAMa 01/58-Su isolate leaves) are significantly different to each other (*P* < 0.05).

	Control				EAMa 01/58-Su			
Volatile compound*	UnSprayed leaf		Sprayed leaf		unsprayed leaf		Sprayed leaf	
**Aldehydes**								
Butanal	33.46 ± 1.46		34.20 ± 1.27		13.57 ± 3.41			
3-methyl-Butanal	516.44 ± 64.81		460.33 ± 160.92					
Pentanal	277.76 ± 51.60	A	100.53 ± 25.31	B				
Hexanal	2821.38 ± 490.76		2258.31 ± 587.30		1665.06 ± 326.19		1630.25 ± 533.92	
3-Methyl-hexanal	29.94 ± 7.12		15.87 ± 2.86					
(E)-2-Pentenal	455.69 ± 59.25	A	603.18 ± 82.50	A	233.83 ± 19.87	B	551.29 ± 198.88	A
Heptanal	42.99 ± 3.99		30.98 ± 7.79		40.24 ± 8.68			
(E)-2-Hexenal	1002.89 ± 154.60		1270.47 ± 218.95		1119.68 ± 151.39		1026.88 ± 37.63	
(Z)-2-Hexenal	40983.33 ± 5616.16		50015.83 ± 8148.49		46673.96 ± 5916.26		40500.00 ± 0.00	
(Z)-4-Heptenal	34.68 ± 6.16	A	26.42 ± 5.31	A				
Octanal	68.40 ± 10.32	A	67.36 ± 11.83	A	50.61 ± 5.44	AB		
(Z)-2-Heptenal	157.52 ± 31.36		31.96 ± 7.42					
(E)-2-Octenal	43.70 ± 3.23		87.15 ± 9.23					
(E)-6-Nonenal	1266.36 ± 185.46	A	337.33 ± 42.74	B	313.90 ± 91.98	B	424.28 ± 77.72	B
(E,E)-2,4-Hexadienal	421.24 ± 38.15	A	585.81 ± 75.03	A	287.69 ± 10.14	B	547.54 ± 107.38	A
Nonanal	364.74 ± 78.17	A	309.59 ± 47.52	A	108.87 ± 23.56	B	287.96 ± 111.29	AB
(E,E)-2,4-Heptadienal	1370.72 ± 198.33	A	1195.76 ± 176.80	AB	1173.54 ± 41.46	B	891.58 ± 230.67	AB
(E)-2-Nonenal	294.45 ± 14.72		26.91 ± 3.61		64.13 ± 6.70		118.00 ± 1.93	
(E,Z)-2,6-Nonadienal	5396.60 ± 983.01		1520.04 ± 536.14		828.50 ± 40.50		181.80 ± 41.26	
(E)-4-Oxohex-2-enal	32.89 ± 5.73	AB	41.30 ± 10.39	A	22.36 ± 1.50	B	15.97 ± 3.31	B
**Alcohols**								
Ethanol					294.30 ± 66.80	A	123.66 ± 18.08	AB
1-Penten-3-ol	2.30 ± 0.57		3.35 ± 0.93					
4-Methyl-1-pentanol					30.11 ± 8.71			
(Z)-3-Hexen-1-ol	4.23 ± 0.33		1.72 ± 0.41		20.25 ± 1.27			
(E)-2-Hexen-1-ol	12.76 ± 2.71	B			58.45 ± 13.77	A	20.78 ± 1.58	AB
1-Octen-3-ol	41.15 ± 4.35	A	11.76 ± 1.03	B	42.31 ± 9.82	AB	15.82 ± 0.72	B
2-Ethyl-1-hexanol	15.91 ± 2.81	A	16.17 ± 1.20	A				
2-Propyl-1-pentanol								
**Ketones**								
6-Methyl-2-heptanone	0.66 ± 0.02		0.46 ± 0.04					
3-Octanone	2.07 ± 0.39		0.65 ± 0.13		9.33 ± 0.79			
6-methyl-5-Hepten-2-one	1.31 ± 0.18	A	0.75 ± 0.20	B	0.61 ± 0.05	B	1.24 ± 0.04	AB
(E,E)-3,5-Octadien-2-one	6.19 ± 3.43		6.02 ± 7.04		7.44 ± 1.06		4.00 ± 1.10	
**Hydrocarbons**								
3-ethyl-1,4-Hexadiene					189.40 ± 75.66			
**Terpenoic derivates**								
β-Cyclocitral	14.79 ± 1.89		16.37 ± 4.05		13.78 ± 3.01		18.76 ± 1.90	
Eucarvone	3.88 ± 1.04		5.49 ± 2.28					
2-Pinen-4-ol	1.77 ± 0.02		1.02 ± 0.15					
β-Citral					3.18 ± 0.38			
Geranial	32.71 ± 0.94		17.89 ± 3.00					
Isomethyl-α-ionone					3.88 ± 1.74			
E-β-ionone					60.64 ± 4.49		44.00 ± 30.59	
2,2,6-Trimethyl-cyclohexanone	3.54 ± 0.39	A	2.39 ± 0.32	B				
β-Ionone					0.54 ± 0.04			
**Phenolic derivates**								
Benzaldehyde	27.00 ± 3.74	A	32.57 ± 3.93	A	14.94 ± 0.90	B	19.89 ± 14.10	AB
Acetophenone	7.28 ± 0.54		7.11 ± 0.45		6.56 ± 0.40		6.29 ± 1.97	
Benzyl alcohol					15.44 ± 0.83			
2-Phenyl-isopranol					3.65 ± 0.71			
Phenol	4.86 ± 0.54		3.82 ± 0.61					
Benzophenone	10.48 ± 0.91		10.36 ± 0.88		14.55 ± 2.04		10.66 ± 4.77	
Toluene	5.62 ± 0.19	B	6.08 ± 0.57	B	5.20 ± 0.79	B	22.34 ± 18.46	A
